# In vitro, genomic characterization and pre-clinical evaluation of a new thermostable lytic *Obolenskvirus* phage formulated as a hydrogel against carbapenem-resistant *Acinetobacter baumannii*

**DOI:** 10.1038/s41598-025-99788-x

**Published:** 2025-05-17

**Authors:** Mahmoud M. Sherif, Neveen A. Abdelaziz, Mohammad Y. Alshahrani, Sarra E. Saleh, Khaled M. Aboshanab

**Affiliations:** 1 Department of Microbiology and Immunology, Faculty of Pharmacy, Ahram Canadian University, Sixth of October City, Giza 12451 Egypt; 2 Institute of Microbiology and Infection, School of Biosciences, University of Birmingham, Dubai, 341799 UAE; 3 Central Labs, King Khalid University, P.O. Box 960, AlQura’a, Abha, Saudi Arabia; 4 Department of Clinical Laboratory Sciences, College of Applied Medical Sciences, King Khalid University, P.O. Box 61413, 9088 Abha, Saudi Arabia; 5 Department of Microbiology and Immunology, Faculty of Pharmacy, Ain Shams University, Cairo, 11566 Egypt

**Keywords:** CRAB, *Acinetobacter baumannii*, Phage, Molecular analysis, Hydrogel, Thermal animal model, Biological techniques, Microbiology

## Abstract

The urgent threat of carbapenem-resistant *Acinetobacter baumannii* (CRAB) necessitates the development of new antimicrobial strategies. Bacteriophage (phage) therapy is one of the most promising alternative strategies that can be implemented to combat multidrug-resistant (MDR) bacterial infections. Herein, an *A. baumannii* phage VB_AB_Acb75 that exhibited lytic activity against 6 CRAB isolates (21.43%) with stability at up to 70 °C, pH 2–12, and high concentrations of organic solvents was isolated and characterized. The transmission electron microscope (TEM) detected a tailed phage with an icosahedral head and contractile tail (myoviral morphotype). The Oxford nanopore sequencing results showed an *A. baumannii* phage genome size of 45,487 bp, a G + C content of 38%, and 42 open reading frames (ORFs). The phylogenetic analysis, ORF, and TEM analysis indicated that *A. baumannii* phage VB_AB_Acb75 belongs to a novel species in the *Obolenskvirus* genus. Furthermore, the phage-loaded Carbopol 940 hydrogel was preclinically evaluated for wound healing effectiveness in the burn-wound animal model infected with the CRAB isolate. The histology findings showed a marked improvement in wound healing through a thick epidermal layer and the formation of well-organized fibrous connective tissue covered by a scab at the site of injury, as well as the ability to eliminate CRAB infection, as compared to the control group. In conclusion, based on in vitro, physicochemical properties, and preclinical findings, the phage-loaded hydrogel is expected to be a promising candidate for clinical evaluation against CRAB-associated skin infections.

## Introduction

According to the World Health Organization (WHO), antimicrobial resistance is one of the most serious risks to global health, accounting for 1.27 million deaths in 2019^1^. The main drivers of the rapid spread of MDR strains include antibiotic abuse and misuse, as well as reduced research and development of novel antibiotics^2^. The Centers for Disease Control and Prevention (CDC) predicts 10 million deaths per year if no new antimicrobial strategies are implemented^3^. In particular, most ESKAPE pathogens (*Enterococcus faecium*, *Staphylococcus aureus*, *Klebsiella pneumoniae*, *Acinetobacter baumannii*, *Pseudomonas aeruginosa*, and *Enterobacter* spp.) are MDR isolates that cause the majority of nosocomial infections worldwide^4^. Studies show that urinary tract infections (UTIs), respiratory tract infections (RTIs), circulatory system infections, and surgical site infections are the most common causes of nosocomial infections^5^.

In North America and certain regions of Europe, the prevalence rate of these infections was reported to be 5%. In certain Asian, Latin American, and African countries, the prevalence rate was approximately 40%^5^. Due to its inherent multi-drug resistance, *A. baumannii* is responsible for a high mortality rate in the intensive care unit^6^.

*A. baumannii* is a Gram-negative pleomorphic coccobacilli, strictly aerobic, non-fermenting, catalase-positive, oxidase-negative, and ubiquitous organism that is usually isolated from natural and healthcare settings^7^. It is one of the most alarming opportunistic nosocomial pathogens that is implicated in a wide array of infections, including hospital-acquired pneumonia, bacteremia, meningitis, UTI, and many life-threatening illnesses^8^. *A. baumannii* holds limitless resistance mechanisms, virulence factors, and a plastic genome, rendering it one of the most resistant bacteria on the earth^9^. Consequently, *A. baumannii* evolved resistance to various antibiotic classes, including colistin, which is considered the last-resort drug to control MDR Gram-negative bacterial infections^10^.

Furthermore, *A. baumannii* exhibited resistance to carbapenems, which were previously considered the drug of choice for their treatment^11^. The primary resistance mechanism is the synthesis of β-lactamases, particularly carbapenem-hydrolyzing enzymes^7^. Furthermore, CRAB caused fatal ventilator-associated pneumonia outbreaks in COVID-19-specific intensive care units^12^. In light of this, both the WHO and the CDC have listed CRAB as an urgent threat necessitating the development of new therapeutic approaches^3,13^.

Phage therapy is one of the most promising alternative strategies that can be implemented to combat MDR bacterial infections^14^. Phages are viruses that can efficiently infect bacterial hosts without alteration of the normal flora^15^. Phages can be classified as lytic (virulent) or temperate (lysogenic)^16^. In lytic phages, the bacterial host is lysed immediately following virion multiplication, and the liberated progeny can infect more bacterial hosts^17^. Temperate phages, on the other hand, either integrate their viral genome with host DNA, resulting in harmless replication, or establish as plasmids. The virus does not become active until host conditions deteriorate, such as nutritional deprivation. At this moment, endogenous phages become active and start the reproductive cycle, causing host cell lysis^18^. In light of this, lytic phages are more suitable for phage therapy.

Although phage therapy has been used in humans for a century, it was largely overlooked during the golden era of novel antibiotic discovery. Today, phage therapy has gained traction as a complementary or alternative strategy to conventional antibiotic therapies because of its high specificity, cost-effectiveness, ease of accessibility, self-replication at the site of infection, and coevolution potential with a bacterial host^19^.

Unfortunately, the number of recovered phages specific to *A. baumannii* is still very minimal despite the earth’s phage diversity^20^. Isolation and characterization of new and more effective *A. baumannii* phages are necessary to be utilized in phage therapy^15^. As a result, the purpose of this study was to isolate and characterize a locally isolated broad-spectrum lytic *A. baumannii* phage in terms of host range, stability, longevity, microscopical, and genomic characteristics, followed by formulation as a hydrogel for preclinical evaluation using a burn-wounded skin animal model infected with a pathogenic CRAB isolate.

## Results

### CRAB clinical isolates and antimicrobial susceptibility test

Twenty-eight MDR isolates were selected for phage recovery from sewage samples. All the isolates were resistant to imipenem (CRAB) and levofloxacin. More than 90% of the isolates were resistant to doxycycline and amikacin. Conversely, only 6% of the isolates exhibited reduced susceptibility to colistin.

### Recovery and screening of phage activity against CRAB clinical isolates

**Fig. 1:**
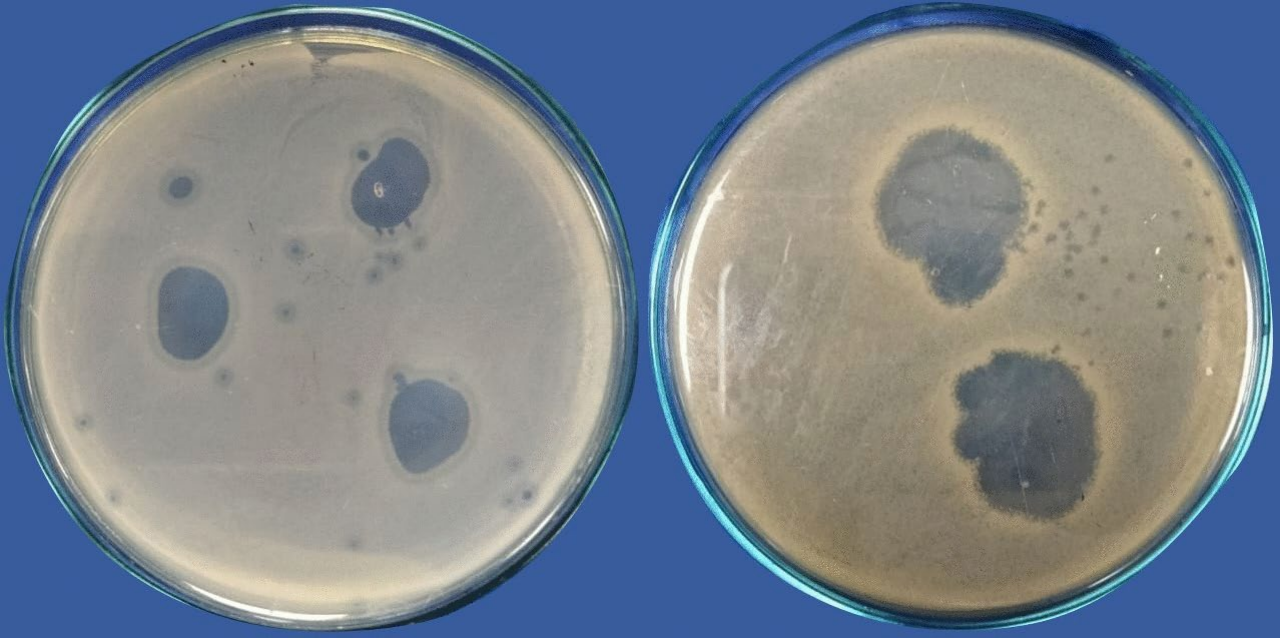
Spot test of the phage lysate that showed the clearest lytic zones on the bacterial lawn.

**Fig. 2:**
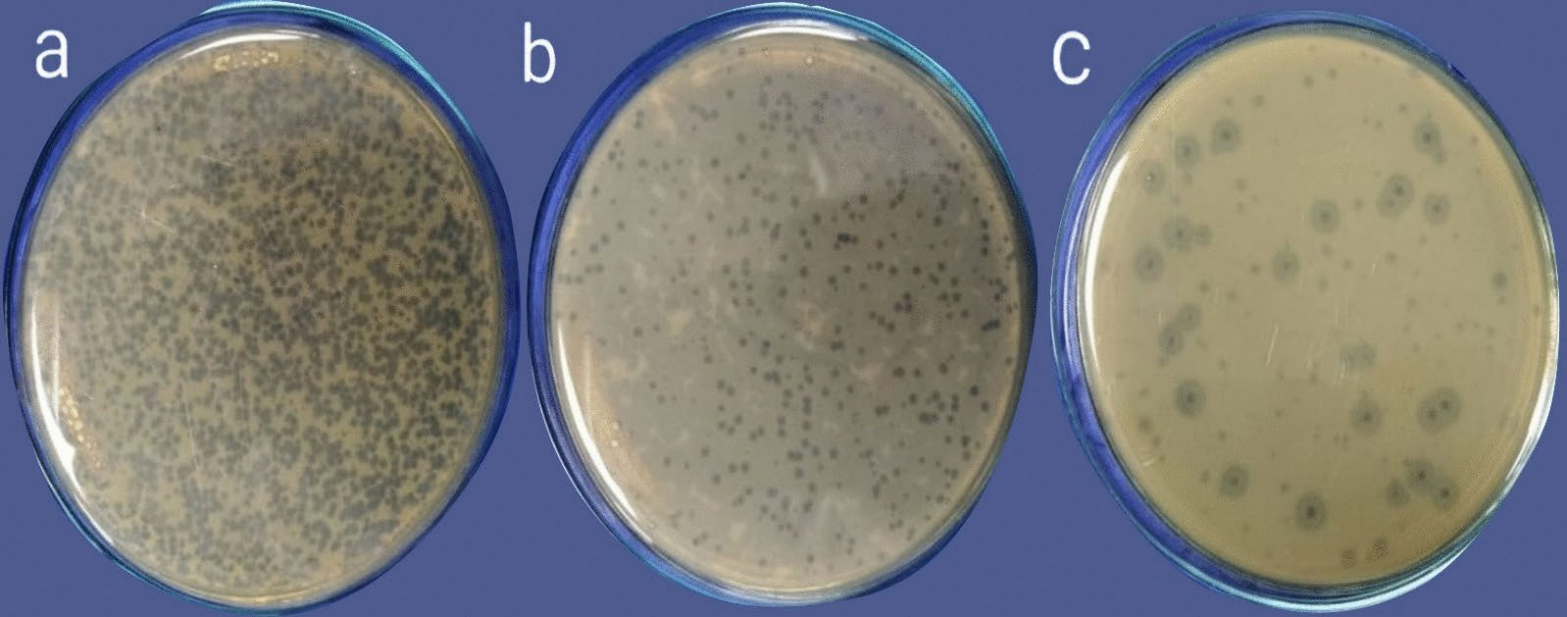
Plaque assay results of *A. baumannii* phage VB_AB_Acb75. (**a**) The dilutions (10^–3^_,_ 10^–4^) exhibited a sheet of interrupted bacterial growth (Net-shaped); (**b**) plaques are too numerous to be counted (TNTC) at dilutions (10^–5^_,_ 10^–6^_,_ 10^–7^); (**c**) well-defined, circular plaques were observed at (10^–8^) dilution.

Different phage lysates were isolated from sewage samples. The isolated lysates were evaluated for their lytic activity against clinical isolates. The phage lysate that showed consistently positive spot tests with the clearest inhibition zone on the bacterial lawn was selected for further studies (Fig. 1). The selected phage lysate showed a reproducible high initial titer (approximately 10^10^ plaque-forming units (PFU)/mL), which was estimated using (Eq. 1) by applying the double-layer overlay agar assay method (Fig. 2). The dilutions (10^–1^_,_ 10^–2^) completely inhibited the growth of the bacterial host.

### Phage host range

Aside from the host strain, the isolated phage lysate exhibited clear lytic activity against five different CRAB clinical isolates. Table S1 shows the antibiogram and carbapenemase genes for the host strain (CRAB1) and the other five susceptible CRAB isolates.

### The TEM imaging

**Fig. 3:**
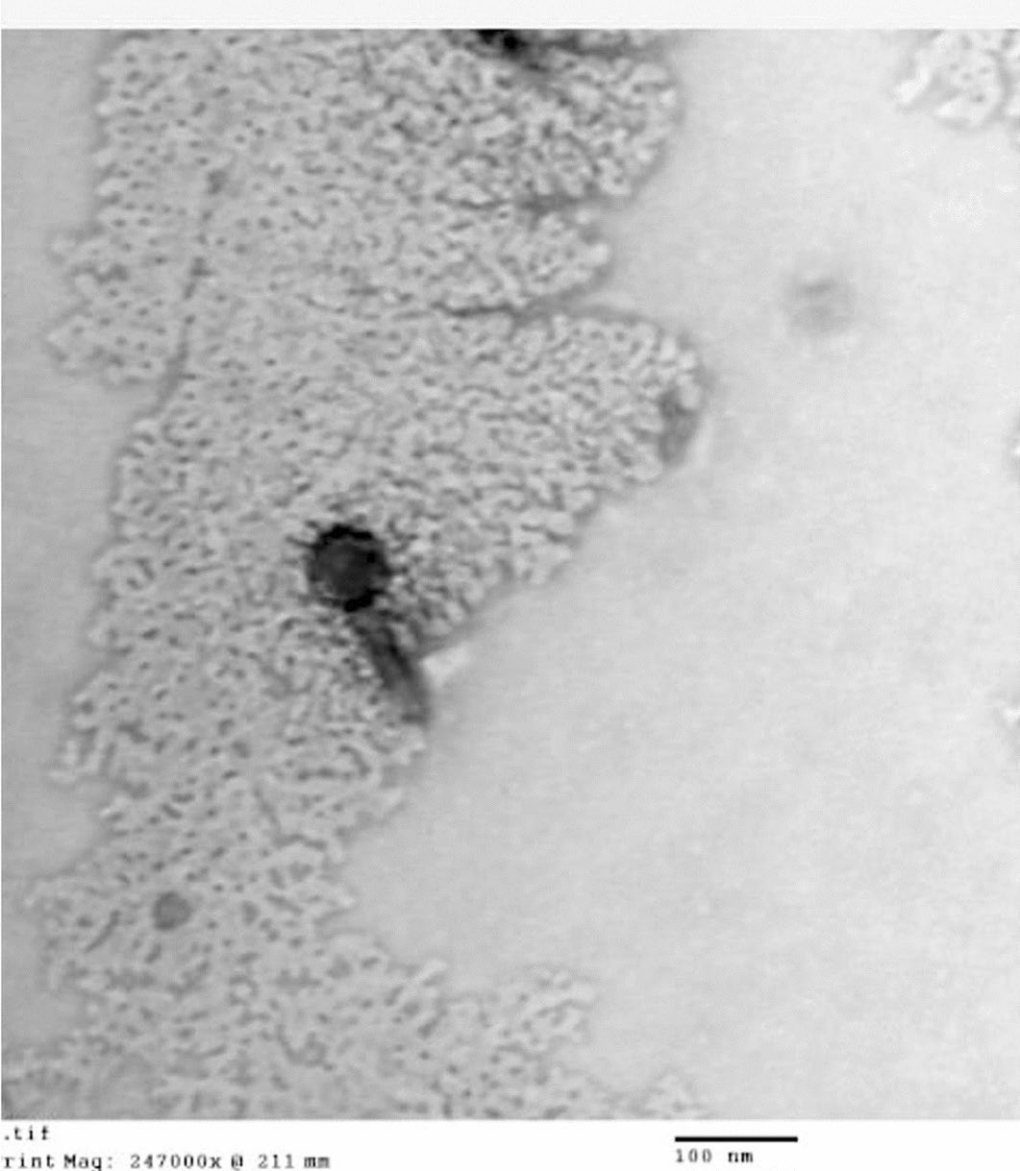
TEM of tailed *A. baumannii* phage VB_AB_Acb75 with an icosahedral head diameter: 56.5 nm; contractile tail length: 86.4 nm. The phage was negatively stained with 2% (w/v) phosphotungstic acid.

The TEM images showed the presence of a tailed phage with an icosahedral head and contractile tail (Fig. 3). The morphological observations along with the data on the “Viral Zone” website and the guidelines provided by the International Committee on Virus Taxonomy (ICTV) suggested that the phage belongs to the myoviral morphotype (formerly family *myoviridae*) of the class *Caudoviricete*s (formerly order *Caudovirales*).

### pH and thermal stability

The phage lysate maintained its lytic activity at pH 2–12. However, at 80 °C, the phage lysate lost its lytic activity, and the lytic spots disappeared completely. As a result, 80 °C was considered the thermal inactivation point.

### Sensitivity to organic solvents

Phage lysate retained its lytic activity at the different concentrations of the three organic solvents (chloroform, ethanol, and isopropyl alcohol); however, the lytic activity partially decreased with 100% ethanol.

### Longevity test

The phage lysate remained infectious at different storage temperatures (4 °C, − 20 °C, and − 80 °C) until the end of the test (90 days).

### Genomic characterization of *A. baumannii* phage VB_AB_Acb75

**Fig. 4:**
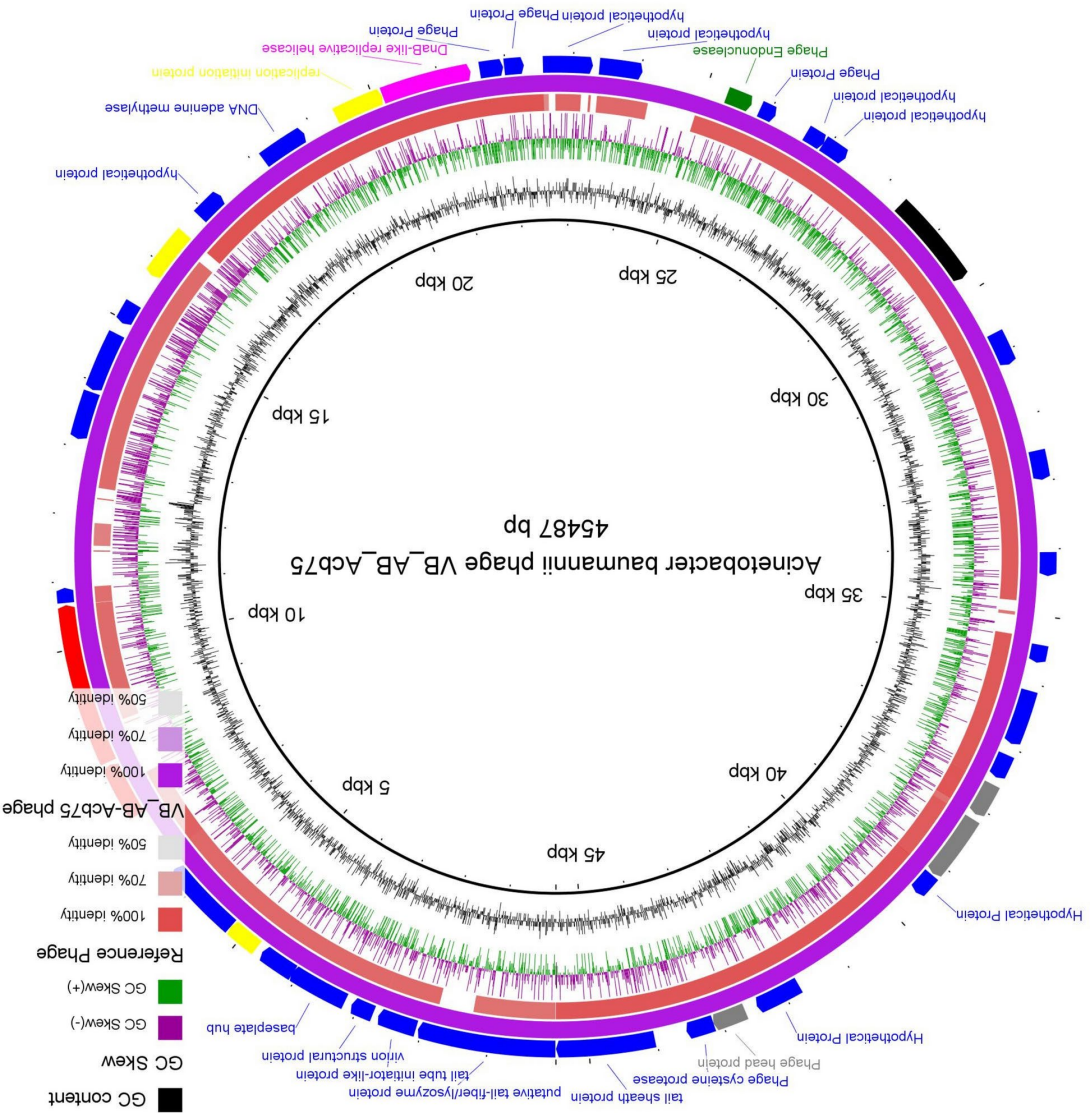
Circular genome map of *A. baumannii* phage VB_AB_Acb75 (NCBI GenBank Accession code, PP717790, purple ring) and the reference phage (red ring; Acinetobacter phage AB1, complete genome; NCBI accession code, NC_042028.1). The phage and hypothetical proteins (blue), terminase protein (black); phage regulatory proteins (yellow), Phage endonuclease or exonuclease (green), Tail protein (red), Capsid protein (gray), Helicase (Fuchsia).

The *A. baumannii* phage VB_AB_Acb75 lysate was sequenced using the qualified R9.4.1 flow cells (FLO-MIN106) instrument, resulting in a 45,487 bp consensus sequence containing 42 Open Reading Frames (ORFs) (38 coded by + frames and 4 coded by − frames). The phages ORFs included 16 coded for structural proteins, 11 coded for non-structural proteins, and 15 coded for hypothetical proteins as shown in Table S2. Table S2 shows the feature annotations and ORF analysis for the *A. baumannii* phage VB_AB_Acb75. The circular genomic map of *A. baumannii* phage VB_AB_Acb75 is shown in (Fig. 4).

### Phylogenetic analysis

The phylogenetic analysis showed that the phage is classified as *Viruses*; *Duplodnaviria*; *Heunggongvirae*; *Uroviricota*; *Caudoviricetes*; *Obolenskvirus*; unclassified *Obolenskvirus* (Fig. S1).

### In vitro antibacterial activity and stability of the phage hydrogel

Both phage lysate alone (positive control) and tested hydrogels (phage-loaded hydrogel) showed consistent positive spot tests with distinct inhibitory zones on the bacterial lawn. In contrast, the control hydrogel (negative control) showed no antibacterial activity (Fig. S2). Furthermore, the phage hydrogel retained its lytic activity after being stored at 4 °C for one month.

### In vivo anti-CRAB activity of the formulated phage hydrogel

#### The survival rate percentage for the examined animal groups

The survival rate % of each group of the examined rats during the trial is shown in (Table S3). All the positive control (E and F) and uninfected groups (A and G), as well as group D (burned, infected, treated with tested hydrogel), showed 100% survival till the end of the experiment (14 days). Group B (infected, untreated) and Group C (infected and treated with vehicle) showed 50% and 75% survival, respectively (Table S3).

#### Histopathological analysis

**Fig. 5:**
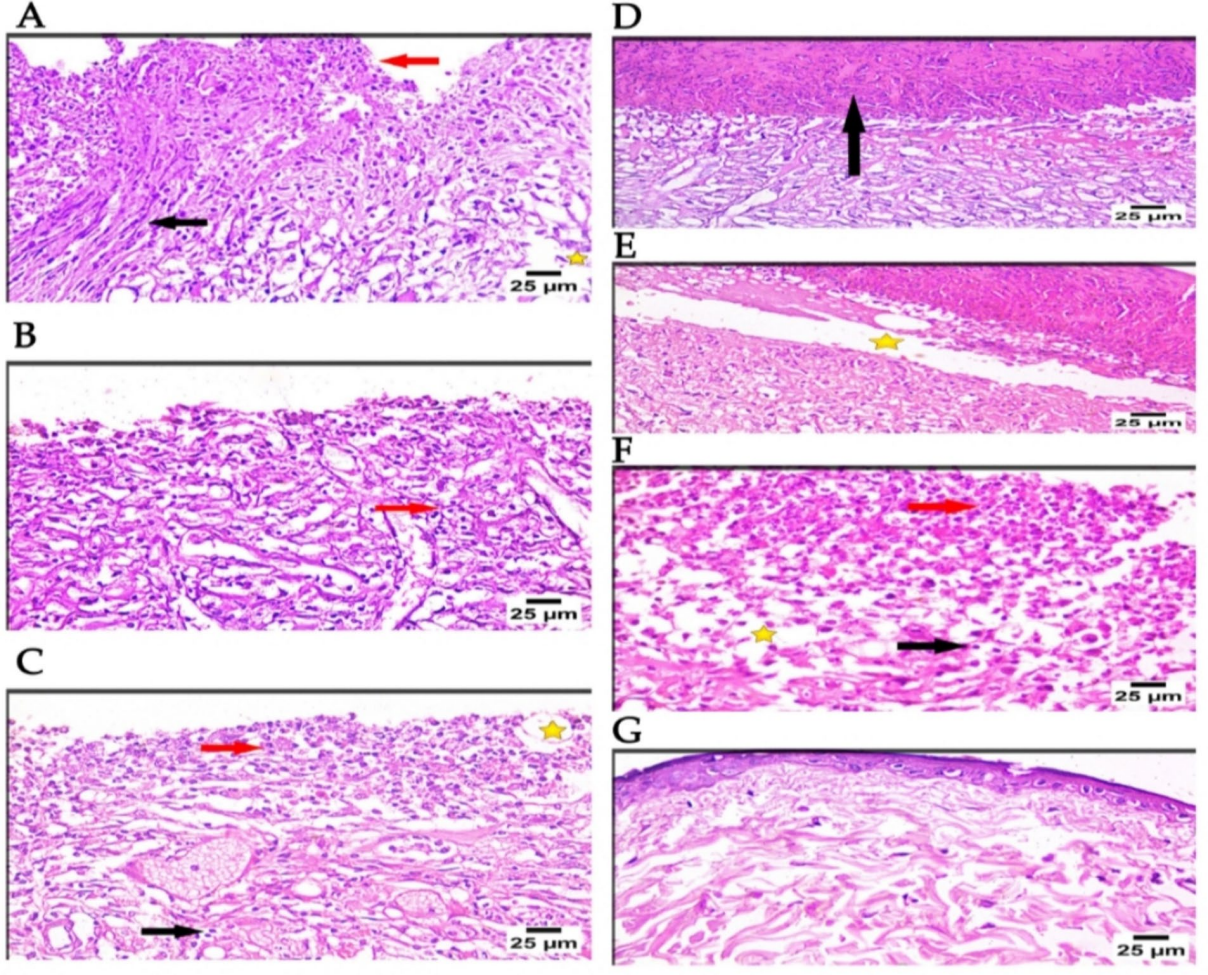
Microscopical examination of histopathological features of skin layers and wound healing process in different groups (H&E; 400X); (**A**) Group A showed sloughing of epidermal layer, coagulative necrosis (red arrow), dispersed collagen fibers (star), and inflammatory cells infiltration in dermis (lymphocytes and neutrophils) as well as fibroblast cells (black arrow), (**B**) Group B showed mild improvement, necrotic tissue with connective tissue formation, dermal edema, and massive infiltration of mononuclear inflammatory cells (red arrow) in the dermal layer, (**C**) Group C showed mild improvement, necrosis with connective tissue formation, interstitial edema (star) with highly infiltration of inflammatory cells mainly neutrophil (red arrow) and lymphocytes (black arrow ) in the dermal layer, (**D**) Group D showed a marked improvement in the form of thick epidermal layer (black arrow) with apparent normally dermis layer, (**E**) Group E revealed moderate improvement, formation of thick epidermal layer with mild separation (star), well organized fibrous connective tissue, and moderate number of mononuclear inflammatory cells infiltration were observed in dermis layer, (**F**) Group F showed mild improvement, dermal edema (star), diffuse necrotic tissue with massive infiltration of inflammatory cells mainly neutrophils (red arrow) and lymphocytes (black arrow ), and connective tissue formation in the dermal layer. (**G**) Group G showed normal skin structure of the dermis & epidermal layers.

Hematoxylin and Eosin (H&E) staining revealed that Group A (control, burned, non-infected, untreated) had sloughing of the epidermal layer, coagulative necrosis, dispersed collagen fibers, and inflammatory cell infiltration in the dermis, primarily lymphocytes and neutrophils, as well as fibroblasts (Fig. 5A), Group B (control, burned, infected, untreated) displayed mild improvement, necrotic tissue with connective tissue development, dermal edema, and significant infiltration of mononuclear inflammatory cells in the dermal layer (Fig. 5B), Group C (control, burned, infected, treated with vehicle) demonstrated minimal improvement, necrosis with connective tissue development, interstitial edema, and a high infiltration of inflammatory cells, primarily neutrophils and lymphocytes, in the dermal layer (Fig. 5C).

**Fig. 6:**
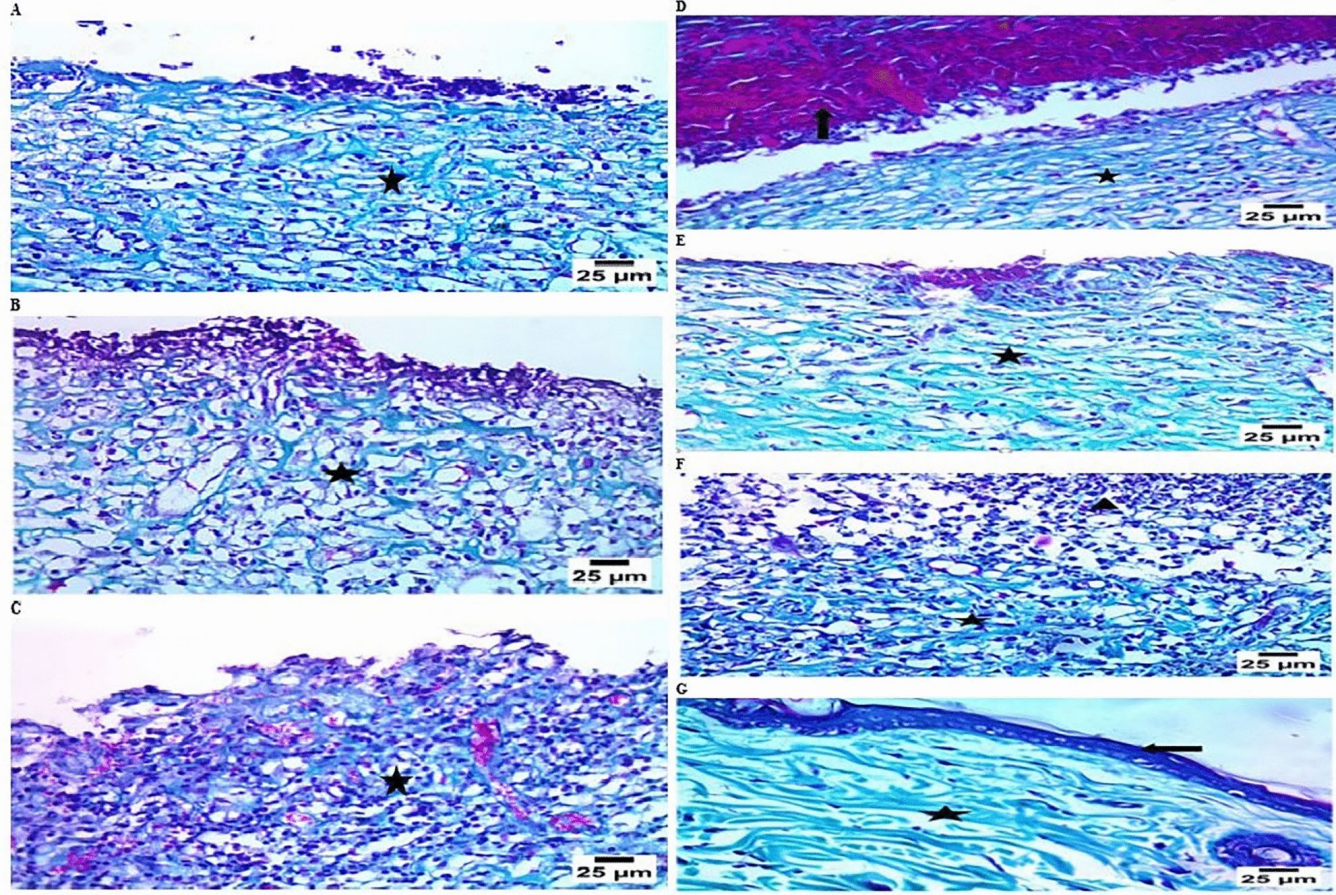
Microscopical examination of Masson’s trichrome satin in different groups (the same paraffin blocks of H&E Sections); (**A**) Group A showed sloughing of the epidermis with fibrous connective tissue formation (star) at the site of injury, (**B**) Group B showed the formation of fibrous connective tissue (star) at the site of injury, (**C**) Group C showed the formation of fibrous connective tissue with infiltration by a high number of inflammatory cells (star) at the site of injury, (**D**) Group D revealed the formation of well- organized fibrous connective tissue (star) covered by scab (arrow) at the site of injury, (**E**) Group E showed the formation of well-organized fibrous connective tissue (star) at the site of injury, (**F**) Group F showed the formation of fibrous connective tissue (star) with infiltration by a high number of inflammatory cells (arrow head) at the site of injury, (**G**) Group G showed the normal histological structure of the epidermis (arrow) and dermis layers (star).

On the other hand, Group D (burned, infected, and treated with the tested phage hydrogel) improved significantly, forming a thick epidermal layer with a normal dermis layer (Fig. 5D), Group E (positive control, burned, infected, treated with gentamycin 0.1%) showed moderate improvement, including the formation of a thick epidermal layer with mild separation, well-organized fibrous connective tissue, and a moderate number of mononuclear inflammatory cell infiltrations in the dermis layer (Fig. 5E), Group F (positive control, burned, infected, and treated with beta-sitosterol) demonstrated minor healing, dermal edema, diffuse necrotic tissue with significant infiltration of inflammatory cells, primarily neutrophils and lymphocytes, and connective tissue development in the dermal layer. (Fig. 5F), Group G showed typical skin structure, including epidermal and dermal layers (Fig. 5G). Furthermore, Masson’s trichrome staining demonstrated that Group A showed sloughing of the epidermis with fibrous connective tissue development at the site of injury (Fig. 6A), Group B showed the development of fibrous connective tissue at the site of injury (Fig. 6B), Group C showed the formation of fibrous connective tissue with infiltration by a large number of inflammatory cells at the site of injury (Fig. 6C), Group D revealed the formation of well-organized fibrous connective tissue covered by scab at the site of injury (Fig. 6D), Group E showed the formation of well-organized fibrous connective tissue at the site of injury (Fig. 6E), Group F showed the formation of fibrous connective tissue with infiltration by a high number of inflammatory cells at the site of injury (Fig. 6F), and Group G showed the normal histological structure of the epidermis and dermis layers (Fig. 6G).

## Discussion

CRAB is regarded as a danger to global public health with few treatment options; thus, WHO designated CRAB as a critical priority for novel antibiotic research and development^13^. In Egyptian hospitals, (26.6–100%) of the isolated *A. baumannii* isolates are CRAB strains, which have a high mortality rate (53.3%)^21^. As a result, implementing alternative therapeutic approaches may be an option for controlling CRAB infections. The recent revival of phage therapy is one of the most promising alternative approaches to control MDR bacterial infections^22^. Despite the advantages of phage therapy, the host-specific infection and the relatively narrow lytic spectrum of the phages limit their potential application.

Phage-antibiotic synergy, phage enzyme separation, phage cocktails, and modified phages are all strategies for overcoming phage therapy constraints^23^. Consequently, the identification and characterization of novel lytic phages is critical for expanding the phage arsenal^22^. The optimal phage for use in phage therapy should have a strict lytic activity to successfully kill its bacterial host. Furthermore, it should be safe to use in humans without causing bacterial hosts to become more resistant or pathogenic^19^.

Plaque morphology differentiates lytic (virulent) phages (clear, transparent plaques) from temperate phages (opaque, turbid plaques)^24^. Additionally, the phage titer (PFU/mL) was estimated using (Eq. 1)^25^. The presence of a translucent halo around plaques produced by *A. baumannii* phage VB_AB_Acb75 indicated the possibility of depolymerase production, which plays a key role in phage adsorption by degrading cap polysaccharide peptides, exopolysaccharide peptides, and lipopolysaccharide peptides, allowing the phage to pass through the bacterial protective barrier and achieve effective sterilization. This effect was observed in various *A. baumannii* phages^26^.

*A. baumannii* phage VB_AB_Acb75 showed lytic activity against six CRAB isolates (21.43%), one of which was resistant to colistin. Similarly, vB_AbaP_W8 and vB_AbaSt_W16 phages from the *Autographiviridae* and *Straboviridae* families displayed lytic activity against 24.14% and 34.48% of CRAB strains, respectively.

On the other hand, vB_AbaSi_W9 phage showed a larger lytic spectrum; however, clear lysis was observed in 10.34% of CRAB clinical isolates^27^. It is worth noting that the primary shortcoming of the previous phage taxonomy classification, the morphology-based family, was an incorrect representation of evolutionary history^28^. The International Committee on Taxonomy of Viruses (ICTV) issued a new taxonomy release (#37), genome-based taxa, in 2022 (https://ictv.global/).

The recent genome-based taxonomy provided a better understanding of the variety and genetic interactions among numerous diverse viruses. The most significant changes in recent phage classification were the removal of the morphology-based families *Myoviridae* (contractile tail), *Podoviridae* (short non-contractile tail), and *Siphoviridae* (long non-contractile tail), as well as the replacement of the order *Caudovirales* by the class *Caudoviricetes*, which grouped all tailed bacterial and archaeal viruses with icosahedral capsids and a double-stranded DNA genome under the *Caudoviricetes* class^29^. The TEM images of *A. baumannii* phage VB_AB_Acb75 showed the presence of a tailed phage with an icosahedral head and contractile tail. Based on its morphological features, the phage is proposed to be a member of the *Caudoviricete* class with a myoviral morphotype.

*A. baumannii* phage VB_AB_Acb75 exhibited lytic activity at pH 2–12, showing high stability in both acidic and alkaline conditions. In prior research, *A. baumannii* phage vB_AbaM_AB3P2, a novel species in the *Obolenskvirus* genus, was stable at pH 2–10 and lost activity at pH 11–12^30^. Furthermore, the *A. baumannii* phage Phab24 showed lytic activity at pH 4–12 and was inactivated at pH values less than 3^31^. Moreover, the Ab_WF01 phage maintained its lytic activity from pH 4 to 12^32^. In terms of thermal stability, *A. baumannii* phage VB_AB_Acb75 retained lytic activity up to 70 °C before becoming completely deactivated at 80 °C. In previous studies, the *A. baumannii* phages Ab_WF01, BUCT628, and pIsf-AB02 were completely deactivated at 70 °C^32–34^. In brief, the *A. baumannii* phage Ab_WF01, a new species of the *Friunavirus* genus, greatly improved the survival rate of CRAB-infected *Galleria mellonella* (from 0 to 70% at 48 h) and mice (from 0 to 60% for 7 days), with significantly less damage to the tissues and clearance of bacteria in the lungs, liver, and spleen in a mouse CRAB infection model^32^. Furthermore, the *A. baumannii* phage BUCT628 rapidly eliminated 56.3% (27/48) of clinical MDR *A. baumannii* isolates. This virulent phage reduced bacterial host cells from 10^8^ CFU/mL to 10^3^ CFU/mL within 30 min with no cytotoxicity to HeLa cells^33^. Additionally, the *A. baumannii* phage pIsf-AB02 showed strong lytic activity against *A.baumannii* infections with a wide range of thermal and pH stability^34^. The pH and thermal stability of *A. baumannii* phage VB_AB_Acb75 are beneficial for practical applications.

Interestingly, *A. baumannii* phage VB_AB_Acb75 retained lytic activity with chloroform, ethanol, and isopropyl alcohol even at high absolute concentrations (100%). Similarly, phage SS3e maintained lytic activity at absolute concentrations of ethanol and isopropyl alcohol, showing that these alcohols had insufficient viricidal effect on the phage^35^. On the other hand, 95% isopropyl alcohol reduced the lytic activity of *A. baumannii* AbTJ phage more than 100% ethanol^36^. Furthermore, phage AHP-1 and Staphylococcus phages Stab20, Stab21, Stab22, and Stab23 remained stable in 100% chloroform^37^.

The genome sequence of *A. baumannii* phage VB_AB_Acb 75 was submitted to the NCBI GenBank database with the accession number PP717790. The *A. baumannii* phage VB_AB_Acb75 genome consists of 45,487 bp of linear dsDNA with 38% G + C content and 42 ORFs. A comparison of this phage genome to nucleotide sequences previously submitted to the NCBI database revealed that *A. baumannii* phage VB_AB_Acb 75 is most similar to *Acinetobacter* phage AB1 (identity, 96.44%; coverage, 88%). A*. baumannii* phage VB_AB_Acb 75 has been classified as a member of a novel species in the *Obolenskvirus* genus based on phylogenetic analysis. The TEM images of *Acinetobacter* phage AB1, a member of the *Obolenskvirus* AB1 species, revealed a siphoviral morphotype (icosahedral head with non-contractile tail). *Acinetobacter* phage AB1’s genome is 45.2 kb of dsDNA with a G + C content of 37.5%^38^.

MDR *A. baumannii* has been associated with recurrent wound infections in burn patients, which can lead to delayed wound healing and the loss of skin grafts^39^. Over the last two decades, MDR *A. baumannii* has become much more common in wound infections linked to combat, according to data from the US military healthcare system. In Iraq, *A. baumannii* was the second most frequently isolated Gram-negative bacterium from clinical infections in U.S. troops^40^. *A. baumannii* is increasingly recognized as a cause of skin and soft tissue infections (SSTIs) associated with combat trauma wounds sustained in war zones such as Iraq and Afghanistan^41^. *A. baumannii* can cause severe necrotizing SSTIs that may progress to bacteremia, especially in patients with comorbidities like trauma. The considerable treatment challenge is amplified by the prevalence of MDR isolates of this bacterium in war-related SSTIs^41^.

Furthermore, the incidence of *A. baumannii*-induced newborn sepsis has risen, with some instances related to a wound infection at the site of the cesarean section^42^. Unfortunately, in many cases, doctors have been obliged to treat *A. baumannii* infections with colistin, a last resort. As a result of a shortage of treatment options, novel medications and tactics for controlling *A. baumannii*-resistant infections have been developed^43^. Several studies have investigated the efficacy of several phages in controlling MDR *A. baumannii* skin infections^44–46^.

Phage biostability and physical stability are the dual challenges facing phage formulation. Liquid formulations of phages that lack controlled delivery are therapeutically insufficient due to the abrupt release and rapid dispersion/elimination of phages from the intended microenvironment. In addition, phages, like other proteins, are unstable in solutions^47^. Because of their customized physical characteristics and biodegradability, hydrogels facilitate the controlled release of phages to target sites, such as wounds^48^. Hydrogels are non-toxic polymers with three-dimensional networks and hydrophilic properties, which allow the gel matrix to contain a high water content^49^. The high water content of the gel matrix provides a favorable environment for biological molecules and live cells, making hydrogels resemble real tissues^50,51^.

Unfortunately, high water content provides a favorable environment for microbial growth, such as yeast, molds, and bacteria, so sterilization of gel formulations by autoclave and then aseptic addition of purified sterile phage lysate using geometric dilution is a method to prepare sterile phage hydrogel formulations^52^. Since the hydrogel will be exposed to high temperatures and pressures during autoclave sterilization, its viscosity, and other physical properties should be evaluated. Chemical preservatives can also be used to maintain aseptic conditions in formulations, with careful consideration in clinical trials due to their antibacterial activity and effect on phage stability^52^.

In our study, we used Carbopol 940, agarose, carboxymethyl cellulose, and xanthan gum to prepare four different phage hydrogel formulations. After steam sterilization (121 °C, 15 min) by autoclave, Carbopol 940 was the most stable gel formulation, retaining its viscosity and physical properties. Our findings were consistent with a previous study^33^. The release of infective phages from hydrogel formulations was qualitatively assessed by spotting formulations on the bacterial lawn, followed by incubation at 37 °C for 24 h^53^. The formation of clear lytic zones indicates phage release from formulations.

Carbopol 940-phage hydrogel showed the clearest and largest lytic zones, indicating an effective phage release profile from the hydrogel. Carbopol 940 hydrogel displayed no antibacterial action against the selected CRAB1 clinical isolate. Furthermore, Carbopol 940-phage hydrogel maintained lytic activity for one month, demonstrating that the phage was stable during the period of the preclinical trial (14 days). Based on the above, Carbopol 940-phage hydrogel was selected for preclinical studies of topical phage-loaded hydrogel. Carbopol 940 is non-toxic, non-irritating, and has a gel-like structure with an appropriate pH for skin application^54^.

A thermal injury model in male rats infected with the CRAB1 isolate was designed to assess the phage hydrogel’s potential to eradicate bacterial infection. Male rats were chosen over females to avoid interference with female hormones in the healing process^55^. The examined animal groups were divided into six control groups and one test group, each with four rats. The histological study of different injured animal groups using H&E and Masson’s trichrome stains revealed varying degrees of tissue damage and healing processes. Staining with H&E showed essential microscopic histological details; however, it cannot provide all the details that we require due to its limited capacity to stain elastic material, reticular fibers, basement membranes, and lipids^56^. Fibrosis can be caused by both physiological and pathological factors, including inflammation, wound healing, and tissue remodeling/repair^57^.

Fortunately, Masson’s Trichrome stain, which uses three stains, is more accurate and can reveal clear images of the collagen fibers compared to H&E stain^57^. As a result, we also used Masson’s trichrome stain, which is more specific in detecting and evaluating the extent and development of fibrosis as a reaction to the healing process in our specimens^56^.

Group A (control, burned, non-infected, untreated) was designed to compare the damage caused by CRAB1 infection in the other groups to this group. Group B (control, burned, infected, untreated) was designed to investigate the virulence and pathogenicity of CRAB1, the persistence of inflammation that slows healing, and the efficacy of topical phages in the burn-wound infection model. Group C (control, burned, infected, treated with hydrogel without phage) was designed to ensure the reproducibility of the data and to attribute the healing activity solely to phage. Group D (burned, infected, and treated with tested hydrogel) was utilized to evaluate the antibacterial activity and healing efficacy of phage-loaded hydrogel. Two positive control groups were designed to act as references for antibacterial activity and wound healing comparison. In group E, gentamicin, a well-known antibiotic, was administered to treat the bacterial-infected wound. The infected burn in group F was treated with beta-sitosterol, which has wound-healing activity. Group G (normal control, intact, non-infected, untreated) was designed to act as a normal control. The H&E stain results revealed a significant improvement in wound healing in group E, in the form of a thick epidermal layer with an apparently normal dermis layer, compared to the other treated groups. Similarly, Masson’s trichrome staining indicated the development of well-organized fibrous connective tissue covered by a scab at the injury site in Group E. Our findings confirmed the efficacy of *A. baumannii* phage VB_AB_Acb 75 in controlling wound-associated infection with the CRAB1 clinical isolate. As a result, further clinical investigations can be conducted to determine the reliability of the specific phage hydrogel for clinical use in humans. Furthermore, the phage can be formulated in various dosage forms and evaluated in preclinical trials to control other resistant *A. baumannii* infections, such as pneumonia, UTI, and bacteremia.

## Conclusion

In this study, we isolated, purified, and characterized a locally isolated lytic *A. baumannii* phage VB_AB_Acb 75 from a hospital sewage sample that exhibited lytic activity against six CRAB isolates. The molecular characterization of the phage suggested that the phage is a member of a new species in the genus *Obolenskvirus* with a siphoviral morphotype. The genome sequencing results showed a phage genome size of 45,487 bp, a G + C content of 38%, and 42 ORFs*. A. baumannii* phage VB_AB_Acb 75 was highly stable at a wide range of temperatures, pH values, and organic solvents. These properties enable the phage to fit various dosage forms and applications. The phage lysate -formulated as a hydrogel- displayed lytic activity against CRAB-infected burn wounds with a marked improvement in wound healing in the burn-wound animal model infected with the CRAB isolate. *A. baumannii* phage VB_AB_Acb 75 is a promising candidate for clinical evaluation as a potential therapeutic option for combating CRAB skin infections.

## Methods

### CRAB clinical isolates identification and antimicrobial susceptibility test

Twenty-eight MDR *A. baumannii* clinical isolates were selected for the isolation of phages from sewage samples. All the selected isolates were CRAB as previously identified and characterized in our lab^58^. In brief, the isolates were identified by detecting the *bla*_OXA-51-like_ gene (intrinsic to *A. baumannii*) by conventional PCR^59^, where *A. baumannii* ATCC 19606 was applied as a positive control. The MIC of the clinical isolates was evaluated against five different antibiotics with different mechanisms of action, namely, colistin, imipenem, doxycycline, levofloxacin, and amikacin by the micro-broth dilution method according to the CLSI guidelines where *E. coli* ATCC 25922 was the reference strain. The isolates were classified as MDR, as previously described^60^.

### Recovery and spot test of phages

To isolate active phages from wastewater samples from Qalioub Specialized Hospital (Qalioub, Egypt) using the enrichment method^61^, 20 mL of clear sewage sample were added to an equal volume of 2X nutrient broth medium (containing 10 mM CaCl_2_), followed by 1 mL of 24-h cultures of the bacterial host (equivalent to 0.5 McFarland). The mixture was then incubated at 37 °C for 48 h with shaking and centrifuged (4000 × *g*) for 10 min, filtered through a 0.22 μm cellulose acetate sterile syringe filter, and stored at 4 °C. The freshly obtained lysates were eventually evaluated for the presence of active lytic phages via spot test^62^. The spot test was performed by adding 100 μL of the bacterial broth in its exponential growth phase to 4 mL of molten soft agar (0.7%), which was overlaid immediately onto regular nutrient agar plates (1.5%). After solidification, 10 μL of the phage lysate was spotted on the top layer of the agar. The plates were incubated in an upright position at 37 °C for 24 h and then evaluated for clear zone formation on the bacterial lawn.

### Plaque formation assay, purification, and propagation

The phage lysate that showed a reliable positive result with the spot test was progressed for the quantitative plaque assay using the double-layer overlay agar assay^63^. The phage lysate was tenfold serially diluted in phosphate buffer saline (PBS), and 100 μL of bacterial suspension (10^8^ CFU/mL) was mixed with 100 μL of each dilution at room temperature for 10 min. This mixture was added to 4 mL of molten soft agar (0.7%), which was poured immediately onto regular nutrient agar plates (1.5%). The plates were incubated at 37 °C for 24 h.

A single plaque was touched with a sterile micropipette tip, inoculated into 2 mL sterile nutrient broth (2X), incubated at 37 °C for 2 h, and purified in five repeated steps by using the double-layer overlay agar assay^64^. The propagation was performed by repeating the phage isolation method 3 times using phage lysate rather than a sewage sample^65^. The obtained high titer of phage lysate was filtered through a 0.22 μm sterile syringe filter, kept at 4 °C, and with sterile glycerol in equal volume at − 80 °C^63^. The phage titer in terms of (PFU/mL) was calculated using the following (Eq. 1)^25^:

1 $$PFU /ml = number\;of\;plaques / (volume\;of\;plaque\;plated\;(ml) \times dilution)$$

### Phage host range

The phage lysate that showed positive results with the spot test with the clearest zone on the bacterial lawn was subsequently processed for host range determination against the remaining 27 CRAB clinical isolates using the spot test as described previously^25^.

### The TEM imaging

The morphologic features of the selected phage lysate were studied using TEM. In brief, 10 μL of purified high titer bacteriophage lysate (approximately 10^10^ PFU/mL) were adsorbed on a carbon-coated 200 mesh copper grid and negatively stained by 2% (w/v) phosphotungstic acid (pH 7.2)^66^. After that, the TEM (JEM-2100, HRTEM, JEOL, Japan) was employed for phage visualization at Nanotech for Photo Electronics Co. (Giza, Egypt).

### pH and thermal stability

The phage stability was evaluated over a broad range of pH (1–12). One milliliter of the phage lysate was added to 1 mL of nutrient broth (2X), which was previously adjusted to a specific pH using 1M hydrogen chloride or 1M sodium hydroxide. After incubation at room temperature for 1 h, phage infectivity was evaluated at the different pH values using a spot test^67^. Moreover, the phage stability was monitored at different thermal conditions (40, 50, 60, 70, 80, and 90 °C) in a previously adjusted water bath for 1 h. After that, an aliquot was aspirated and spotted for infectivity^25^. The pH and thermal stability experiments were conducted in triplicates.

### Effect of organic solvents

Chloroform, ethanol, and isopropyl alcohol at 10%, 30%, 50%, 70%, and 100% v/v dilutions were used to test the sensitivity of the recovered phage to organic solvents. The phage lysate was mixed with each concentration (1:1 ratio) and left at room temperature for 1 h. The effect was evaluated via spot test^68^. The tests were repeated in triplicates.

### Longevity test

Aliquots of phage lysate were kept at 4 °C, − 20 °C, and − 80 °C for 90 days. The aliquots were evaluated for lytic activity at various time intervals using spot assays, as previously described^25^.

### Molecular analysis of the phage

#### Phage DNA isolation and library preparation

A purified high titer of phage lysate (approximately 10^10^ PFU/mL) was used for DNA extraction using a phage DNA isolation kit (Cat. No. 46800, Norgen Biotek Corp., Canada) according to the manufacturer’s instructions. The extracted DNA was quantified using a Qubit 4 fluorometer (Thermo Fisher Scientific, USA). A rapid sequencing kit (SQK‐RAD004, Oxford Nanopore Technologies, Oxford, UK) was used for library preparation according to the manufacturer’s instructions.

#### Oxford nanopore sequencing and assembly

The molecular sequencing was performed by loading the prepared library on qualified R9.4.1 flow cells (FLO‐MIN106, Oxford Nanopore Technologies). MinKNOW software ver. 23.11.5 (Oxford Nanopore Technologies) was used for data acquisition. The Dorado basecall server ver.7.3.9 (Oxford Nanopore Technologies) on AWS EC2 g4dn.xlarge was used for the conversion of MinION™ sequence reads into fastq files. Kraken2 and Recentrfuge were used for the classification and visualization of fastq files, respectively. The de novo assembly was performed using Flye, and the draft assembly was polished three times using Medaka. The final consensus sequence was analyzed as previously reported^69^. The assembled phage sequence was submitted to the NCBI GenBank database (Accession Number PP717790).

#### Phylogenetic analysis of the phage

The generated raw sequencing data via the Oxford Nanopore Technology platform was obtained in FASTQ format. We applied DIAMOND to annotate and analyze the functional potential of the dataset. DIAMOND is a high-speed alignment tool that can be utilized to align the sequences against the NCBI non-redundant (nr) protein database^70^. To perform taxonomic classification and construct a phylogenetic tree, the resulting output was imported into MEGAN (MEtaGenome Analyzer)^71^. To assign reads to taxa and generate a phylogenetic tree that visualizes the taxonomic relationships in the dataset, MEGAN utilizes a Lowest Common Ancestor (LCA) algorithm. This approach allowed the representation of the microbial diversity present in the sample accurately.

#### Formulation of topical phage-loaded hydrogel

Carbopol 940 hydrogel (1%) was prepared by dissolving 1 g of Carbopol powder in 100 mL of distilled water and mixing with a magnetic stirrer at room temperature for 1 h at 8000 rpm. The pH was adjusted to 7 ± 0.2 by dropping triethanolamine to jellify the polymer^72^. Subsequently, 9 mL of hydrogel was poured into glass vials, which were subjected to steam sterilization (121 °C, 15 min) by autoclave^73^. After that, 1 mL of the phage lysate with an initial titer (approximately 10^9^ PFU/mL) was aseptically added to the sterilized hydrogel vials (9 mL) to obtain a final titer of approximately 10^8^ PFU/mL^44^. The phage-loaded hydrogel was stored at 4 °C before use.

#### In vitro antibacterial activity and stability of the phage hydrogel

The antibacterial activity of the phage lysate, tested phage hydrogel, and vehicle hydrogel (negative control) was qualitatively assessed using the spot test^74^. In brief, 10 μL of different samples were dropped on the bacterial lawn (0.5 McFarland) on a nutrient agar plate, incubated at 37 °C for 24 h, and tested for clear zone formation on the bacterial lawn. To determine stability, the phage-loaded hydrogel was refrigerated at 4 °C for one month before being tested for lytic activity maintenance on the bacterial lawn using a spot test.

### Preclinical evaluation of topical phage-loaded hydrogel

#### Animal model

Twenty-eight adult male Wistar albino rats weighing approximately 200–220 g were used throughout the experiment. All rats were kept in open cages and fed an antibiotic-free diet with free access to water. They were housed at a constant temperature of 25 °C, which was adjusted by air conditioning.

#### Experimental animal wound model

The burn wound infection with CRAB clinical isolate was induced by using a pre-heated rectangular metal bar (1** × **2 cm and 1 mm thickness) to induce burns on the dorsal side after the shaving of the burn site. Then, the infection was induced by applying 0.5 mL of bacterial suspension (10^8^ CFU/mL) to the burn wound site^75^. The examined animal groups were categorized into six control groups and one test group (each of 4 animals), as previously described^76^ and displayed in Table S3.

#### Treatment

The tested hydrogel, control hydrogel, Garamycin®, and Mebo® were all applied topically to the infected burn two hours after infection. After that, the treatment was administered twice every day for 14 days. Dead animals were removed from the groups and used only to estimate survival rates. The rats were euthanized via cervical dislocation following intraperitoneal administration of a mixture of 60 mg/kg ketamine and 10 mg/kg xylazine for anesthesia. The dorsal skin at the wound site was promptly extracted when the animals were slaughtered and preserved in a 10% formalin solution for histological investigation^76^.

#### Histological analysis

Skin samples were fixed in 10% neutral buffered formalin for 72 h before being decalcified in 10% formic acid, trimmed, rinsed in water, dehydrated in increasing grades of ethyl alcohol, clarified in xylene, and embedded in paraffin. A rotatory microtome was used to generate 5 μm thick tissue sections in the central zones of wound samples, revealing the various skin layers^75^. After that, Tissue sections were stained with H&E and Masson’s trichrome stains according to standard procedures, and the tissues were evaluated for histological alterations in a blind manner^77^.

## Supplementary Information

Supplementary Information.

## Data Availability

The authors declared that the data supporting the findings of this study are available within the article and its supplementary information file. The final assembled sequence of the phage genome was annotated and submitted to the NCBI GenBank database under the accession code, PP717790. https://www.ncbi.nlm.nih.gov/nuccore/PP717790.
