# Supplementary material for: In vitro, genomic characterization and pre-clinical evaluation of a new thermostable lytic Obolenskvirus phage formulated as a hydrogel against carbapenem-resistant Acinetobacter baumannii
Source: Sci Rep. 2025 May 17;15:17149. doi: 10.1038/s41598-025-99788-x (PMC12085604; doi:10.1038/s41598-025-99788-x)
Supplement: Supplementary file 1 — Supplementary Information. [file 41598_2025_99788_MOESM1_ESM.docx]

**Table S1** Summary for susceptibility pattern, MIC of IPM, and genotypic detection of carbapenemase genes of CRAB isolates.

| **Isolate number** | **Susceptibility patterns** | | | | | **MIC of IPM (µg/ml)** | **Carbapenemase genes** |
| --- | --- | --- | --- | --- | --- | --- | --- |
|  | **CL** | **IPM** | **DOX** | **AK** | **LEV** |  |  |
| CRAB1 | S | R | R | R | R | 32 | *bla* _NDM_, *bla* _VIM_ |
| CRAB2 | S | R | R | R | R | 512 | *bla* _VIM_ |
| CRAB3 | S | R | R | R | R | 64 | *bla* _NDM_, *bla* _VIM_ |
| CRAB4 | R | R | R | R | R | 256 | *bla* _oxa-23_ |
| CRAB5 | S | R | R | R | R | 256 | *bla* _oxa-23_ |
| CRAB6 | S | R | R | R | R | 128 | *bla* _VIM_ |

CL, colistin; IPM, imipenem; DOX, doxycycline; AK, amikacin; LEV, levofloxacin; MIC, minimum inhibitory concentration; *bla* _NDM:_ New Delhi metallo-beta-lactamase (group B beta-lactamase); *bla* _VIM:_ Verona integrin associated metallo-beta-lactamase (group B beta-lactamase); *bla* _oxa-23:_ Oxacillinase beta lactamase (group D beta-lactamase).

**Table S2** Feature annotations and open reading frame (ORF) analysis of Acinetobacter baumannii phage VB_AB_Acb75

| **ORF Number** | **Feature ORF name** | **Strand** | **Interval range** |
| --- | --- | --- | --- |
| 1 | putative tail-fiber/lysozyme protein | **+** | 9..2057 |
| 2 | tail tube initiator-like protein | **+** | 2089..2673 |
| 3 | virion structural protein | **+** | 2758..3099 |
| 4 | baseplate hub | **+** | 3209..4099 |
| 5 | baseplate spike | **+** | 4080..4625 |
| 6 | Tail sheath initiator protein | **+** | 4728..5225 |
| 7 | putative baseplate J-like protein | **+** | 5222..6406 |
| 8 | structural protein | **+** | 6620..7246 |
| 9 | tail fiber protein | **+** | 7368..8213 |
| 10 | tail fiber protein | **+** | 8258..10657 |
| 11 | hypothetical protein | **+** | 10692..10913 |
| 12 | exonuclease of Acinetobacter phage | **-** | complement (13119.13835) |
| 13 | RecT-like ssDNA annealing protein | **-** | complement (13871.14770) |
| 14 | hypothetical protein | **-** | complement (14946.15281) |
| 15 | transcriptional regulator | **-** | complement (15769.16560) |
| 16 | hypothetical protein | **+** | 16891..17325 |
| 17 | DNA adenine methylase | **+** | 18147..18857 |
| 18 | replication initiation protein | **+** | 19390..20133 |
| 19 | DnaB-like replicative helicase | **+** | 20139..21482 |
| 20 | Phage Protein | **+** | 21618..21965 |
| 21 | Phage Protein | **+** | 21982..22272 |
| 22 | hypothetical protein | **+** | 22553..23290 |
| 23 | hypothetical protein | **+** | 23386..24024 |
| 24 | Phage Endonuclease | **+** | 25315..25701 |
| 25 | Phage Protein | **+** | 25843..26088 |
| 26 | hypothetical protein | **+** | 26612..26908 |
| 27 | hypothetical protein | **+** | 26905..27294 |
| 28 | putative terminase large subunit | **+** | 28355..29773 |
| 29 | phage portal protein | **+** | 30704..31213 |
| 30 | hypothetical protein | **+** | 32544..32981 |
| 31 | hypothetical protein | **+** | 34049..34402 |
| 32 | hypothetical protein | **+** | 35421..35687 |
| 33 | prohead core protein protease | **+** | 36112..36939 |
| 34 | head maturation protease | **+** | 37113..37481 |
| 35 | capsid stabilizing protein | **+** | 37596..38102 |
| 36 | major capsid protein | **+** | 38184..39176 |
| 37 | Hypothetical Protein | **+** | 39200..39550 |
| 38 | hypothetical protein | **+** | 41755..42444 |
| 39 | Phage head protein | **+** | 42630..43133 |
| 40 | Phage cysteine protease | **+** | 43130..43543 |
| 41 | tail completion protein | **+** | 43570..44034 |
| 42 | tail sheath protein | **+** | 44024..45487 |

**Table S3.** The survival rate percentage of the animals examined

| Group | Group Description | survival% |
| --- | --- | --- |
| a | Control, burned, non-infected, untreated | 100 |
| B | Control, burned, infected, untreated | 50 |
| C | Control, burned, infected, treated with vehicle (control hydrogel) | 75 |
| D | Burned, infected, treated with tested hydrogel | 100 |
| E | Positive Control, burned, infected, treated with beta sitosterol (Mebo^®^) | 100 |
| F | Positive control, burned, infected, treated with gentamycin 0.1% | 100 |
| G | Normal control, intact, non-infected, untreated | 100 |


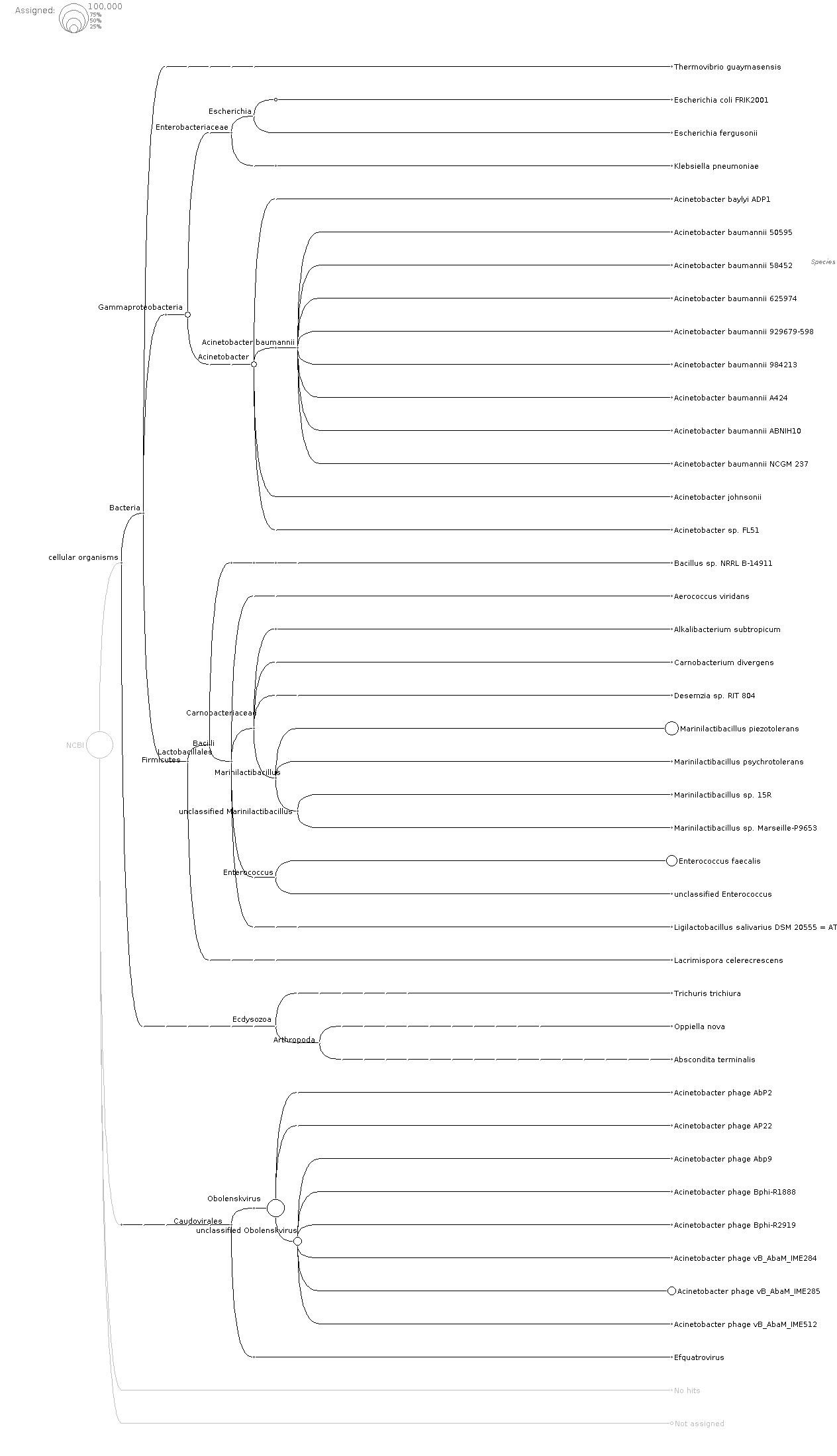


**Fig. S1** Phylogenetic tree of A. baumannii phage VB_AB_Acb75.


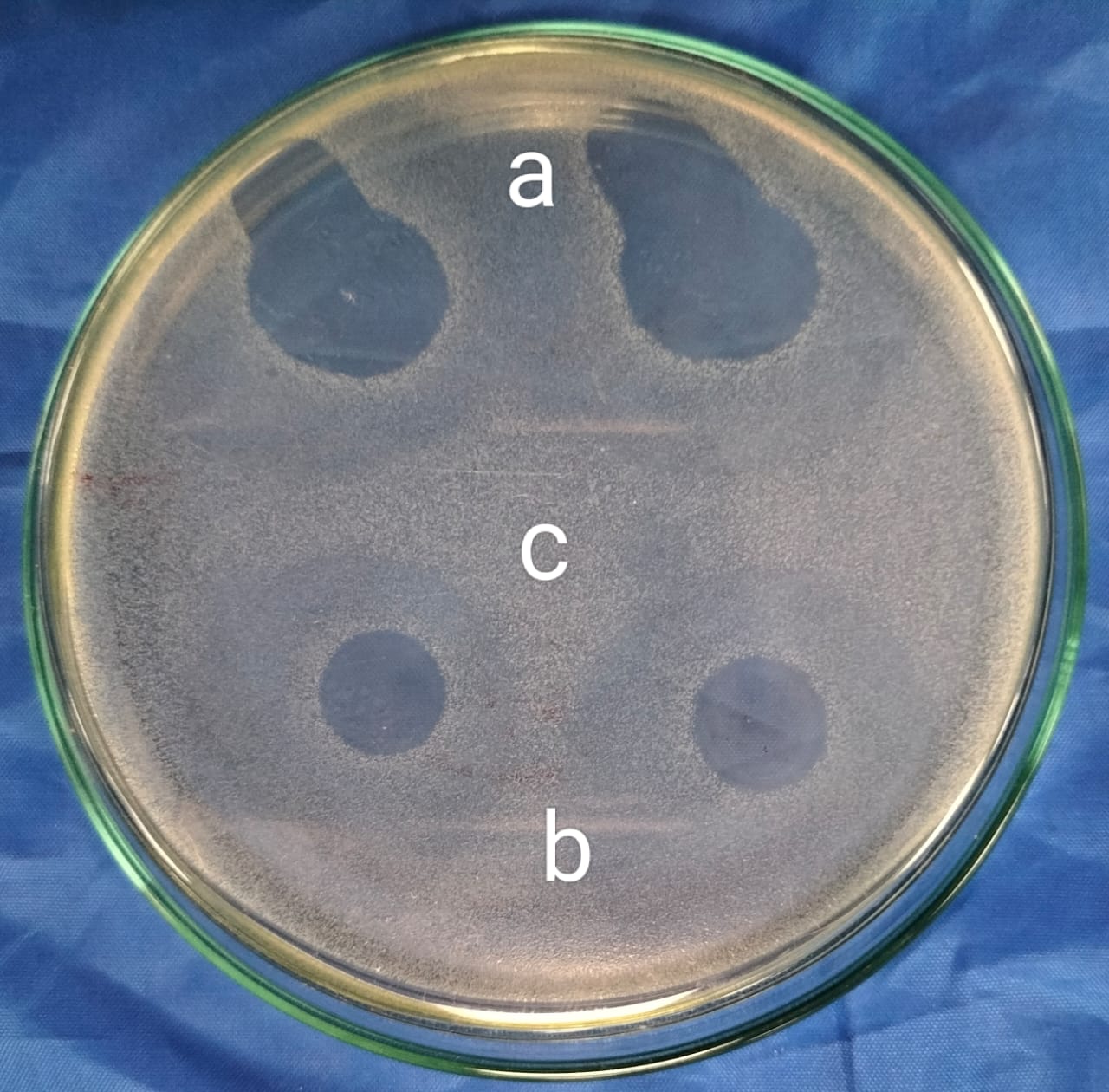


**Fig. S2** *In vitro* lytic activities of A. baumannii phage VB_AB_Acb75 against carbapenem resistant *Acinetobacter baumann*ii clinical isolate (CRAB1): (a) the phage lysate exhibited clear lytic spots; (b) phage-loaded hydrogel showed clear lytic spots similar to zones observed with phage lysate alone; (c) formulated Carbopol 940 hydrogel (1%).showed no antilytic activity
